# Disulfide Bond Formation and *N-*Glycosylation Modulate Protein-Protein Interactions in GPI-Transamidase (GPIT)

**DOI:** 10.1038/srep45912

**Published:** 2017-04-04

**Authors:** Lina Yi, Gunes Bozkurt, Qiubai Li, Stanley Lo, Anant K. Menon, Hao Wu

**Affiliations:** 1 Department of Biological Chemistry and Molecular Pharmacology, Harvard Medical School, Boston, MA 02115, USA; 2 Program in Cellular and Molecular Medicine, Boston Children’s Hospital, Boston, MA 02115, USA; 3 Weill Cornell Graduate School of Medical Sciences, New York, NY 10065, USA; 4 Department of Biochemistry, Weill Cornell Medical College, New York, NY, 10065, USA

## Abstract

Glycosylphosphatidylinositol (GPI) transamidase (GPIT), the enzyme that attaches GPI anchors to proteins as they enter the lumen of the endoplasmic reticulum, is a membrane-bound hetero-pentameric complex consisting of Gpi8, Gpi16, Gaa1, Gpi17 and Gab1. Here, we expressed and purified the luminal domain of *Saccharomyces cerevisiae (S. cerevisiae*) Gpi8 using different expression systems, and examined its interaction with insect cell expressed luminal domain of *S. cerevisiae* Gpi16. We found that the N-terminal caspase-like domain of Gpi8 forms a disulfide-linked dimer, which is strengthened by *N-*glycosylation. The non-core domain of Gpi8 following the caspase-like domain inhibits this dimerization. In contrast to the previously reported disulfide linkage between Gpi8 and Gpi16 in human and trypanosome GPIT, our data show that the luminal domains of *S. cerevisiae* Gpi8 and *S. cerevisiae* Gpi16 do not interact directly, nor do they form a disulfide bond in the intact *S. cerevisiae* GPIT. Our data suggest that subunit interactions within the GPIT complex from different species may vary, a feature that should be taken into account in future structural and functional studies.

The biosynthesis of glycosylphosphatidylinositol (GPI)-anchored proteins (such as acetylcholinesterase, folate receptor, prion protein, Thy-1 and the neural cell adhesion molecule (NCAM)) is critical for normal cell growth and perturbed in human cancers and a number of genetic diseases. The latter include paroxysmal nocturnal hemoglobinuria, an acquired hemolytic disease that is caused by a defect in the first step of GPI biosynthesis in multipotent hematopoietic human stem cells. Genetic abrogation of GPI biosynthesis results in embryonic lethality in mammals. GPI-anchored proteins are critical for the viability of a parasitic protozoa and fungi, and the GPI assembly pathway has been validated as a therapeutic target for protozoal and fungal diseases. GPI transamidase (GPIT), the enzyme that attaches GPI anchors to protein, is a poorly understood membrane-bound hetero-pentameric complex. Genes encoding three GPIT subunits have been recently identified as oncogenes, raising the possibility that the GPI pathway may provide a novel target for cancer treatment^1^,^2^,^3^.

The five subunits of GPIT are PIG-K/Gpi8, GAA1/Gaa1, PIG-T/Gpi16, PIG-S/Gpi17 and PIG-U/Gab1, in mammals and yeast, respectively^4^,^5^,^6^. (Fig. 1A) The subunits are conserved in eukaryotes, from protozoa, yeast to mammals, either by sequence or membrane topology, and all five subunits are needed for the transamidation reaction^4^,^5^,^7^,^8^. PIG-K/Gpi8 has been experimentally shown to be the catalytic subunit within the complex^9^,^10^,^11^, and each of the other four subunits has a proposed functional role in the transamidation reaction. GAA1/Gaa1, is a polytopic membrane protein that has been suggested to promote the binding of mature GPI-anchor to the proprotein^5^,^12^,^13^. The lumenal domain of PIG-T/Gpi16 is predicted to have a β-propeller structural fold^14^, and it is thought to directly interact with PIG-K/Gpi8^15^. As a result, PIG-T/Gpi16 is proposed to facilitate the access of substrate proteins to the active site of PIG-K/Gpi8. Furthermore, several genetic diseases have been linked to PIG-T/Gpi16^16^,^17^. PIG-S/Gpi17 is speculated to play role in proprotein selectivity and/or may fix the hydrophobic tail of the proprotein in the transition state^14^. PIG-U/Gab1, another multi-membrane spanning protein, is suggested to recognize long chain fatty acids of the GPI lipid mainly because its sequence contains a motif found in mammalian and yeast fatty acid elongases^5^,^12^,^18^.

Even though each of the GPIT subunits is postulated to have a unique function in the transamidation reaction, experimental tests of these assignments are hampered by the lack of structural information and a robust *in vitro* GPIT activity assay. Currently, only low-resolution structure mapping of partial luminal domains of PIG-K/Gpi8, GAA1/Gaa1 and PIG-S/Gpi17 are available through the small-angle X-ray scattering (SAXS) method^19^. While working towards reconstituting the *S. cerevisiae* GPIT complex for structural studies we made unexpected findings on the properties of *S. cerevisiae* Gpi8 and *S. cerevisiae* Gpi16 with regards to disulfide pairings and *N*-glycosylation in the Gpi8-Gpi16 binary complex. Here we present these findings and discuss their implications for future structural studies on the complex.

## Results

### *Saccharomyces cerevisiae* GPI-Transamidase Complex is Most Likely Dimeric

**Figure 1:**
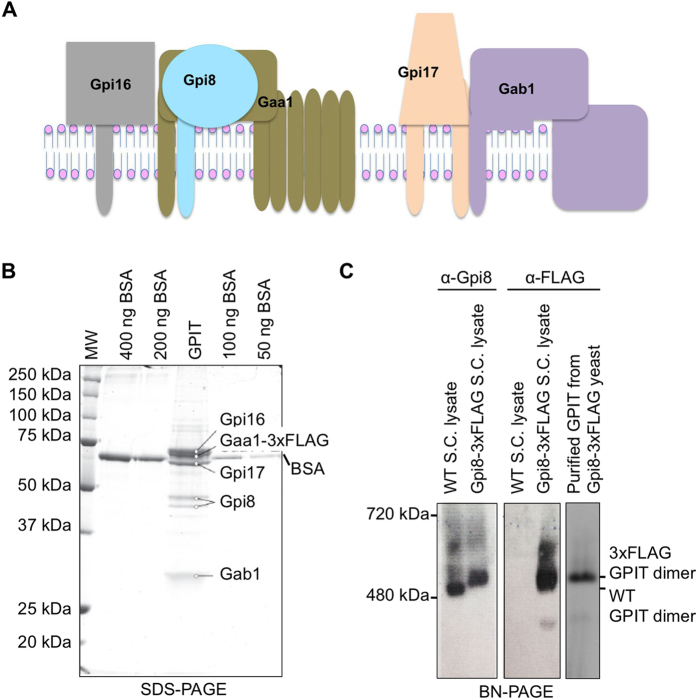
Immunopurification of endogenous *S. cerevisiae* GPIT complex. (**A**) Schematic organization of *S. cerevisiae* GPIT. (**B**) SDS-PAGE analysis of the immunopurified *S. cerevisiae* GPIT complex from a *S. cerevisiae* strain expressing Gaa1-3xFLAG. A digitonin extract of microsomes was precleared with IgG-agarose before being incubated with anti-FLAG agarose. Bound GPIT complexes were eluted with 3xFLAG peptide and analyzed by 10% reducing SDS-PAGE. Lanes 2, 3, 5 and 6 correspond to 400, 200, 100 and 50 ng BSA and the GPIT sample is in lane 4. *N-*glycosylation of Gpi8 results in the observed doublet. (**C**) Endogenous *S. cerevisiae* GPIT complexes on a 6% Blue Native PAGE (BN-PAGE) followed by Western blotting. The Western blots indicate that *S. cerevisiae* GPIT runs as a dimer on a BN-PAGE.

Towards reconstituting a GPI-transamidase (GPIT) complex for structural studies, we first investigated the interaction among the five GPIT subunits under physiological conditions. A triple tandem FLAG (3xFLAG) tag was individually engineered into the C-terminus of 4 subunits of the endogenous GPIT proteins to create 4 different strains of *Saccharomyces cerevisiae (S. cerevisiae).* In each case, expression of the tagged subunit was from the genomic locus of the native open reading frame and under natural regulation. As GPIT is essential for yeast viability, the ability to generate these strains indicated that C-terminal tagging did not affect function. We extracted the endogenous GPIT complex from each strain using 1% digitonin. Immunopurified *S. cerevisiae* GPIT from the four different 3xFLAG-tagged strains showed similar results on SDS-PAGE analysis, and the purified GPIT from the strain expressing Gaa1-3xFLAG on a 10% SDS-PAGE is shown in Fig. 1B. The full-length, membrane bound endogenous *S. cerevisiae* Gpi8 has been shown to possess multiple *N*-glycosylation sites^9^, resulting in the observed doublet. As shown for human PIG-U (equivalent to *S. cerevisiae* Gab1)^4^, the *S. cerevisiae* Gab1 migration position is much lower than its calcualted molecular weight (Fig. 1B).

To characterize the endogenous GPIT complex, we first ran wild type (WT) and 3xFLAG-tagged *S. cerevisiae* lysates using blue native polyacrylamide gel electrophoresis (BN-PAGE) followed by either anti-Gpi8 or anti-FLAG Western blotting. Compared to regular native PAGE, BN-PAGE allows separation of the complexes based on the molecular mass while still preserving the intact protein complexes^20^,^21^. Given that GPIT likely consists of an equi-stoichiometric complex of Gpi8, Gpi16, Gaa1, Gpi17 and Gab1^15^,^18^,^22^, the predicted monomeric molecular mass of *S. cerevisiae* GPIT is ~290 kDa. *S. cerevisiae* GPIT migrated on BN-PAGE as high molecular mass bands between the molecular weight markers at 480 kDa and 720 kDa (Fig. 1C), suggesting that it is dimeric. The WT GPIT migrated somewhat faster than the 3xFLAG reconstituted counterpart, likely because of the highly charged nature of the FLAG tag sequence. Anti-FLAG purified GPIT ran homogenously on BN-PAGE at a position similar to the major band in the 3xFLAG reconstituted *S. cerevisiae* lysates (Fig. 1C). Human GPIT was also reported to be large from a velocity gradient with a sedimentation coefficient of ~17S^23^.

### Dimerization of the Caspase-like Domain of the Luminal Region of *S. cerevisiae* Gpi8

**Figure 2:**
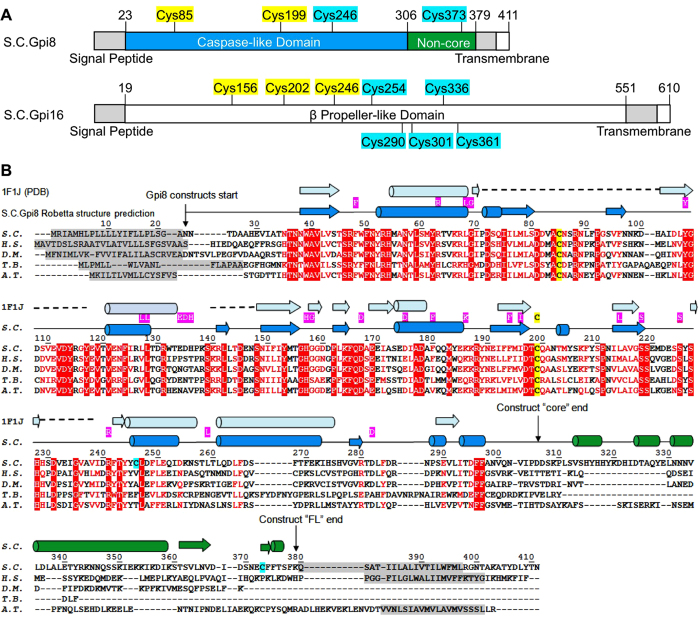
Sequence and structural analysis of *S. cerevisiae* Gpi8 and *S. cerevisiae* Gpi16 proteins. (**A**) Domain organization of *S. cerevisiae* Gpi8 and *S. cerevisiae* Gpi16. Locations of conserved Cys residues are highlighted in yellow and of non-conserved Cys residues in cyan. (**B**) Sequence and structural analysis of *S. cerevisiae* Gpi8. Sequence alignment of Gpi8 from different species, *S.C., H.S. (Homo sapiens), D.M. (Drosophila melanogaster), T.B. (Trypanosoma brucei), and A.T. (Arabidopsis Thaliana*). Residues identical among the different species are shown in white on a red background. Conserved, but not identical, residues are shown as red letters. Secondary structures predicted in the *S. cerevisiae* Gpi8 model from Robetta are shown above the protein sequences in blue. Because Gpi8 has a caspase-like domain, we aligned *S. cerevisiae* Gpi8 with caspase-7 (PDB code 1F1J)^29^ and the corresponding secondary structure from caspase-7 are shown in pale blue. Identical residues between *S. cerevisiae* Gpi8 and caspase-7 are shown in white with a pink background. Dashed lines represent deletions in caspase-7 sequence relative to *S. cerevisiae* Gpi8.

The luminal region of Gpi8 contains a previously identified caspase-like domain^24^ followed by a non-core domain (Fig. 2). The caspase-like domain within the luminal region of Gpi8 has previously been expressed in the reducing environment of *Escherichia coli (E. coli*) cytoplasm^24^. Gpi8 contains four Cys residues in its luminal domain, with three in the caspase-like domain (Fig. 2), and localizes in the oxidizing environment of the endoplasmic reticulum (ER) lumen. Thus, we decided to express Gpi8 as a secreted protein in insect cells to promote proper folding and disulfide pairing in the oxidizing environment of the secretory pathway. To keep the oxidizing environment, we also purified the recombinant Gpi8 under a non-reducing condition in the absence of dithiothreitol (DTT).

**Figure 3:**
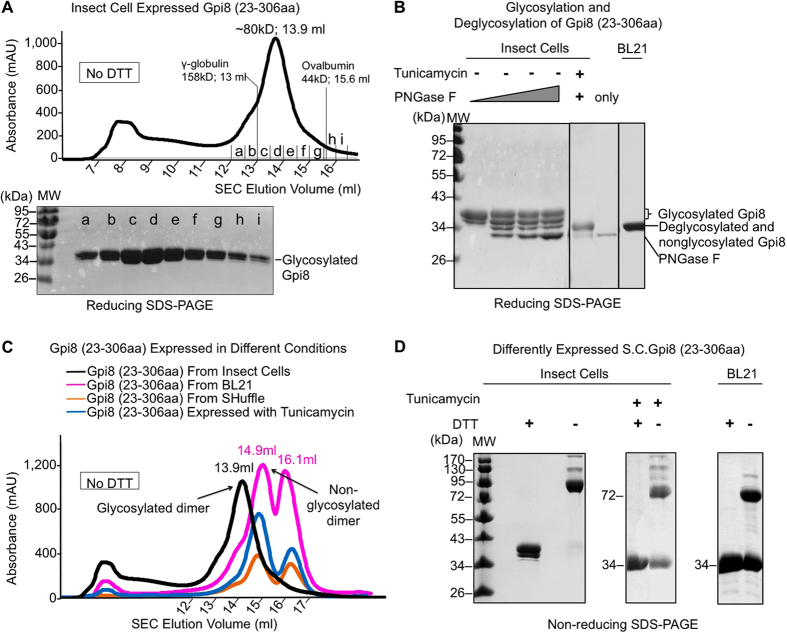
Biochemical characterization of the caspase-like domain of *S. cerevisiae* Gpi8 (23-306aa). (**A**) Size exclusion chromatography (SEC) profile and SDS-PAGE of insect cell expressed *S. cerevisiae* Gpi8 (23-306aa) under a non-reducing purification condition. (**B**) Glycosylated, deglycosylated and nonglycosylated Gpi8 are shown on reducing SDS-PAGE. When *S. cerevisiae* Gpi8 was expressed in the presence of 2.5 mg/l tunicamycin, *N*-glycosylation was inhibited. (**C**) Superposition of the SEC profiles of different *S. cerevisiae* Gpi8 (23-306aa) samples: insect cells-expressed (black), insect cell-expressed in the presence of 2.5 mg/l tunicamycin (blue), *E. coli* BL21-expressed (pink), and *E. coli* Shuffle-expressed (orange). (**D**) Non-reducing SDS-PAGE of differently expressed *S. cerevisiae* Gpi8 (23-306aa).

Expressing the caspase-like domain of *S. cerevisiae* Gpi8 (23-306aa) as a secreted protein in insect cells resulted in an appreciable yield (Fig. 3A). In contrast to the mostly monomeric state of the same Gpi8 construct expressed previously in the *E. coli* BL21 cytoplasm^24^, the insect cell expressed Gpi8 appeared to form stable dimers in gel filtration (Fig. 3A). In addition, our SDS-PAGE analysis showed that insect cell expressed Gpi8 has two bands around 47 kDa implying that the protein was probably posttranslationally modified during insect cell expression (Fig. 3B). There are two predicted *N*-glycosylation sites in *S. cerevisiae* Gpi8 (23-306aa). We first confirmed that the insect cell expressed Gpi8 was *N*-glycosylated during the expression by treating the protein with endoglycosidases such as PNGase F (Fig. 3B). The treatment generated a triplet of bands with the two upper bands most likely corresponding to glycosylated forms and the lowest band being completely deglycosylated. We then expressed *S. cerevisiae* Gpi8 in insect cells in the presence of 2.5 mg/L tunicamycin, which serves to block addition of *N*-acetylglucosamine to dolicholphosphate in the first step of *N*-linked oligosaccharide formation. The purified *S. cerevisiae* Gpi8 expressed in the presence of tunicamycin showed a migration location on SDS-PAGE similar to the lowest band after deglycosylation (Fig. 3B). Interestingly, its size exclusion chromatography (SEC) profile indicated mostly dimers, but with significant amount of monomers (Fig. 3C), suggesting that glycosylation somehow enhanced dimerization.

Given that *E. coli* BL21 expressed *S. cerevisiae* Gpi8 (23-306aa) reported previously exhibited mostly monomeric species, we attempted bacterial expression using three different bacterial cell lines, *E. coli* BL21, *E. coli* Rosetta-gami 2, and *E. coli* SHuffle. Rosetta-gami 2 harbors mutations in both the thioredoxin reductase (*trxB*) and glutathione reductase (*gor*) genes, while SHuffle constitutively expresses disulfide isomerase DsbC. In theory, both may enhance disulfide bond formation in the *E. coli* cytoplasm. Expression of *S. cerevisiae* Gpi8 in *E. coli* BL21 and *E. coli* SHuffle both resulted in almost equal amounts of dimers and monomers while Rosetta-gami 2 showed very low expression (Fig. 3C). Because of the similar expression results from *E. coli* BL21 and *E. coli* SHuffle, we only used *E. coli* BL21 in further experiments. Re-run of the insect cell and *E. coli* expressed dimer peaks of Gpi8 on SEC showed that they stayed as dimers (Supplementary Fig. S1), suggesting that the dimer peaks cannot easily dissociate into monomers. In contrast, the monomer peak of *E. coli* BL21 expressed Gpi8 eluted at both dimer and monomer positions (Supplementary Fig. S1), suggesting that dimers could be formed from monomers. Collectively, these data suggest that Gpi8 can form dimers when expressed either in the more reducing environment in the *E. coli* cytoplasm or the more oxidized environment in the insect cell secretary pathway.

### *S. cerevisiae* Gpi8 (23-306aa) Can Form Homo-dimers Through an Interchain Disulfide Bond

*S. cerevisiae* Gpi8 (23-306aa) contains three Cys residues at sites 85, 199 and 246, respectively (Fig. 2). C199 is the predicted catalytic residue. Because of the different degrees of dimerization in the different redox environments, we asked whether the dimers could be linked via disulfide bonds. To elucidate this, we run non-reducing SDS-PAGE in the absence of DTT in the SDS sample buffer. Strikingly, we observed a major band at around 70 kDa representing the dimeric state of the insect cell expressed Gpi8 (Fig. 3D), indicating linkage by interchain disulfide bond in the dimer. Insect cell expressed Gpi8 in the presence of the glycosylation inhibitor tunicamycin showed a mixture of dimers and monomers on non-reducing SDS-PAGE (Fig. 3D). Similarly, *E. coli* expressed Gpi8 also showed a mixture of monomers and dimers, consistent with the SEC profile (Fig. 3C,D). In comparison, when DTT was added to the SDS sample buffer, only monomers were observed on SDS-PAGE for all Gpi8 samples (Fig. 3D).

**Figure 4:**
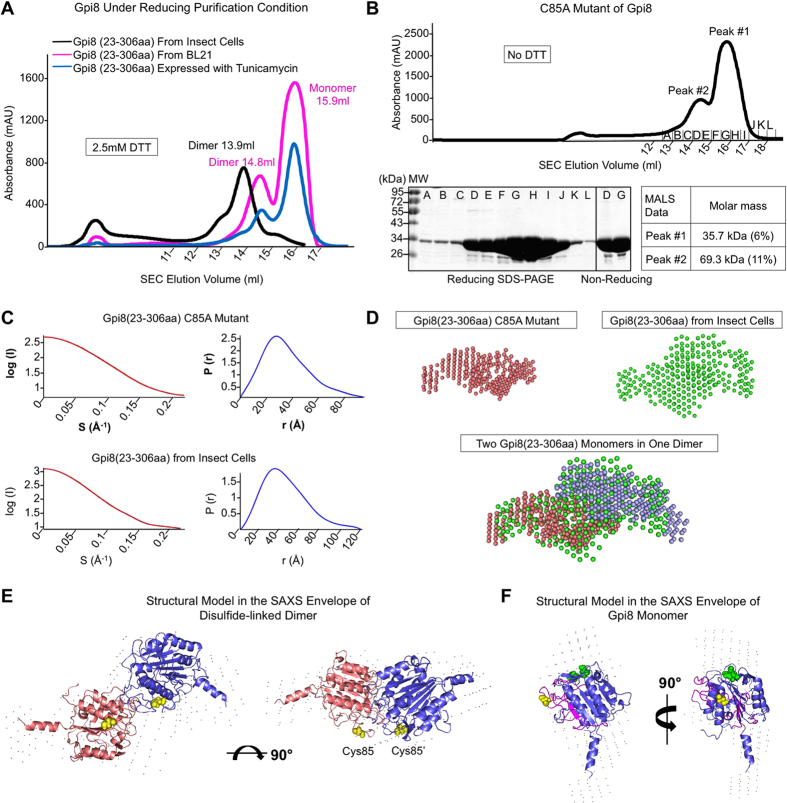
The importance of residue C85 and small-angle X-ray Scattering (SAXS) analysis on *S. cerevisiae* Gpi8 caspase-like domain. (**A**) Size exclusion chromatography (SEC) profile of different versions of *S. cerevisiae* Gpi8 (23-306aa) under reducing conditions. The insect cells-expressed version is shown in black. The insect cell-expressed sample in the presence of 2.5 mg/l tunicamycin is shown in blue. The *E. coli* BL21-expressed version is shown in pink. (**B**) Purification of the C85A mutant of *S. cerevisiae* Gpi8 (23-306aa), showing size exclusion chromatography (SEC) profile, MALS analysis of the C85A mutant under reducing conditions, and SDS-PAGE of C85A under reducing and non-reducing conditions. (**C**) SAXS scattering curves and pair-distance distribution functions P(r) of the *S. cerevisiae* Gpi8 (23-306aa) C85A monomer and insect cell-expressed WT *S. cerevisiae* Gpi8 (23-306aa) dimer. (**D**) Top: The individual SAXS bead models of Gpi8 C85A monomers and WT dimers. Bottom: Two bead models from *S. cerevisiae* Gpi8 (23-306aa) monomer are fitted into the bead model from the *S. cerevisiae* Gpi8 (23-306aa) dimer. (**E**) Two copies of the Robetta structural model of *S. cerevisiae* Gpi8 (23-306aa) are positioned into the *S. cerevisiae* Gpi8 (23-306aa) dimer SAXS envelope with the Cys85 residue from each molecule facing and interacting with each other. (**F**) The same Robetta structural model of *S. cerevisiae* Gpi8 (23-306aa) is within the *S. cerevisiae* Gpi8 (23-306aa) C85A monomer SAXS envelope. The residues in magenta represent insertions in *S. cerevisiae* Gpi8 relative to the caspase-7 structure. Active site Cys199 and His157 are colored in Green. Cys85 is in yellow.

To further elucidate the relationship between disulfide bonds and dimer formation, we tested the effect of adding DTT to the protein purification buffers. For both insect cell expressed Gpi8 in the presence of tunicamycin and *E. coli* expressed Gpi8, the SEC profiles shifted so that a majority of the proteins came out in monomeric peaks (Fig. 4A). These data suggest that disulfide bond formation is critical for the stability of the observed dimers. Consistently, previously reported recombinant *S. cerevisiae* Gpi8 caspase-like domain expressed^®^ in *E. coli* and purified in the presence of DTT is also mostly monomeric^24^. However, insect cell expressed, fully glycosylated Gpi8 did not dissociate into monomers in the presence of DTT (Fig. 4A). We suspect that the *N*-glycans may have shielded the access of DTT to the disulfide bond at the dimer interface. This is supported by the complete dissociation into monomers on reducing SDS-PAGE under the denaturing condition (Fig. 3D).

### Cys85 of *S. cerevisiae* Gpi8 is Responsible for Disulfide Bond Formation

Having confirmed the hypothesis that *S. cerevisiae* Gpi8 (23-306aa) can form disulfide-linked homo-dimers, we individually mutated the three Cys residues within the protein construct (Fig. 2A) and investigated its SEC profiles under both reducing and non-reducing purification processes. While the C199A and C246A mutations did not change the dimer to monomer ratios in comparison with the WT (Supplementary Fig. S2), the C85A mutation caused a shift to mostly monomeric positions on the SEC profile even in the absence of DTT (Fig. 4B). Non-reducing SDS-PAGE showed that the dimer peak did not contain disulfide-linked species (Fig. 4B). Furthermore, multi-angle light scattering (MALS) measurement confirmed the dimeric and monomer molecular masses of the peaks (Fig. 4B). Re-run of the monomer peak on SEC in the absence of DTT showed only the monomer peak (Supplementary Fig. S2). Unlike the dimer peak from either insect cell or *E. coli* expressed WT Gpi8 which stayed as dimers, the dimer peak of C85A exhibited a mixture of dimers and monomers in the SEC re-run, suggesting equilibrium between dimers and monomers in the absence of disulfide linkage (Supplementary Fig. S2).

Because *E. coli* expressed C85A mutant and insect cell expressed WT Gpi8 gave high quantities of stable monomer and dimer respectively, we used small-angle X-ray scattering (SAXS) to gain further structural insights on factors affecting dimerization. Both Guinier plots of the proteins show absences of aggregation, so we processed the data and calculated the pair-distance distribution function P(r) (Fig. 4C). We generated *ab initio* molecular envelopes for both the C85A monomer and the WT dimer. The SAXS envelope of the C85A mutant appears to be of a tubular shape with one end larger than the other. The dimer envelope matched well with two monomer envelopes (Fig. 4D), suggesting the validity of the calculations. Previously, Toh and colleagues^19^ performed SAXS analysis on *S. cerevisiae* Gpi8 (24-337aa) expressed from *E. coli*, showing a similar monomeric envelope for Gpi8, whereas we report here the envelope and model for the dimeric species as well.

Because Gpi8 has been predicted to have a caspase-like fold^24^, we used the protein structure prediction server Robetta^25^ to obtain structural information on the luminal region of *S. cerevisiae* Gpi8. Five independent structural models were generated. The caspase-like domain (23-306aa) showed remarkable similarity among the five models, with pairwise RMSDs between 0.7 Å to 1.2 Å (Supplementary Fig. S3). Within the caspase-like domain, the conserved Cys85 and the active site Cys199 are exposed in the model while Cys246 is completely buried. Because insect cell expressed Gpi8 (23-306aa) is bonded via a disulfide linkage to form homo-dimers, we placed two Gpi8 (23-306aa) Robetta models into the Gpi8 (23-306aa) SAXS envelope with Cys85 from each molecule facing and interacting with each other (Fig. 4E). Such a placement also positioned the two glycosylation sites adjacent to the dimerization interface (Fig. 4E), which could have played a role in dimer stabilization and in protecting the disulfide bond from DTT. The model fitting in the dimer envelope was then transferred to the C85A monomer envelope using the superposition between the dimer and monomer envelopes, with reasonable agreement (Fig. 4D,F).

### The Effects of the Non-core Domain on Cys85 Disulfide Bond Formation

**Figure 5:**
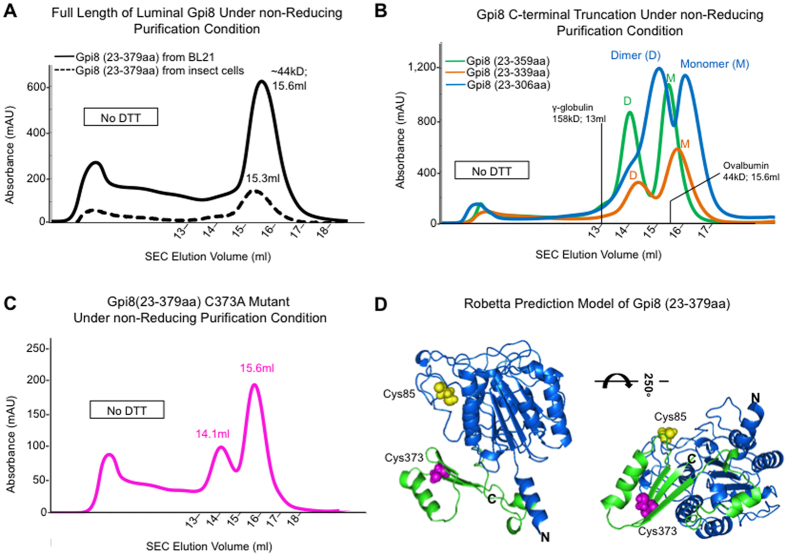
Biochemical characterization of the luminal domain of *S. cerevisiae* Gpi8 (23-379aa), its mutant C373A and its truncations under non-reducing conditions. (**A**) Full-length luminal region of Gpi8 is mostly monomeric. SEC profiles of insect cell-expressed (solid) and *E. coli*-expressed (dashed) *S. cerevisiae* Gpi8 (23-379aa) under non-reducing conditions. (**B**) C-terminal truncated versions of the luminal domain of *S. cerevisiae* Gpi8 (23-379aa) contain both dimers and monomers under non-reducing purification condition. SEC profiles of Gpi8 C-truncations, *S. cerevisiae* Gpi8 (23-359aa), *S. cerevisiae* Gpi8 (23-337aa) and *S. cerevisiae* Gpi8 (23-306aa) under non-reducing purification condition are respectively shown in green, orange and blue. (**C**) SEC profile of *S. cerevisiae* Gpi8 (23-379aa) C373A mutant under a non-reducing purification condition. (**D**) Structural Model of the full-length *S. cerevisiae* Gpi8 luminal region. Robetta structural model of full-length luminal region of *S. cerevisiae* Gpi8 (23-379aa) is shown as a ribbon. Cys85 is in yellow and Cys373 is in magenta.

The luminal domain of *S. cerevisiae* Gpi8 (23–379) contains a non-core domain of around 80 residues after the conserved caspase-like domain that has no recognizable sequence similarity to known domains (Fig. 2). We expressed full-length luminal domain of Gpi8 (23-379aa) in both *E. coli* cytoplasm and as a secretary protein in insect cells. Surprisingly, the SEC profiles even in the absence of DTT showed that both full-length luminal Gpi8 proteins are mostly monomers (Fig. 5A), unlike the caspase-like domain alone. We tested if the presence of the non-core domain somehow inhibited dimerization using deletion studies. Indeed, progressive deletion of parts of the non-core domain seemed to correlate with re-appearance of the dimer peak (Fig. 5B), supporting the hypothesis.

Because Cys85 is a main factor in dimer formation for the caspase-like domain, we wondered if Cys85 is paired internally in the full-length luminal Gpi8 construct. Only one Cys residue is present in the non-core domain of *S. cerevisiae* Gpi8, Cys373 (Fig. 2). When we mutated Cys373 to Ala within the full-length luminal construct, the SEC profile under the non-reducing purification condition appeared as a mixture of dimers and monomers (Fig. 5C). Therefore, the conserved Cys85 must have once again involved in the disulfide-linked dimer formation in the absence of intramolecular pairing with Cys373. In the Robetta models that we generated, the non-core region preferentially forms a structure with a central three-stranded β-sheet flanked by α-helices (Fig. 5D). The beginning of the non-core domain is apparently loopy and flexible, which created different relative orientations between the caspase-like and non-core domains in the different models. In one of the models, the locations of Cys85 and Cys373 are juxtaposed for potential disulfide bond formation (Fig. 5D).

### *S. cerevisiae* Gpi8 Does Not Form a Disulfide-linked Hetero-dimer with *S. cerevisiae* Gpi16

Our data indicated that Cys85 has a high propensity for forming inter- or intra-molecular disulfide bond in *S. cerevisiae* Gpi8. In the intact human GPIT complex, previous studies have also implicated that PIG-K and PIG-T are linked together through a disulfide bond in their luminal domains, Cys92 of PIG-K and Cys182 of PIG-T^15^, equivalent to Cys85 of *S. cerevisiae* Gpi8 and Cys202 of *S. cerevisiae* Gpi16. Collectively, these data all suggest that the conserved Cys85 of *S. cerevisiae* Gpi8, or its equivalent in human PIG-K, is a critical residue for the structure of Gpi8 itself or of the GPIT complex. However, the possibility of alternative disulfide pairings relating to Cys85 also causes confusion in its physiological role within the GPIT complex.

**Figure 6:**
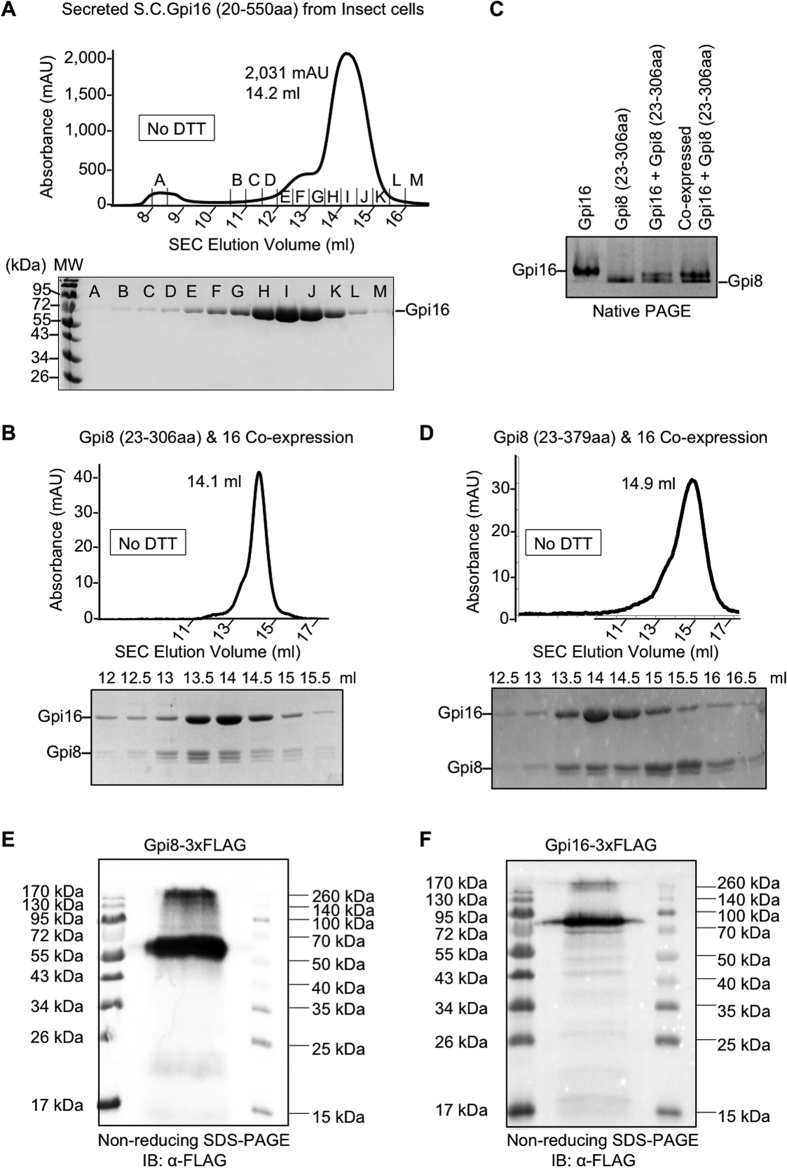
*S. cerevisiae* Gpi8 and *S. cerevisiae* Gpi16 luminal domains do not interact via a disulfide bond. (**A**) The SEC profile and SDS-PAGE of the full-length luminal *S. cerevisiae* Gpi16 (20-550aa) expressed in insect cells. (**B**) SEC profile and SDS-PAGE analysis on co-expressed *S. cerevisiae* Gpi16 (20-550aa) and *S. cerevisiae* Gpi8 (23-306aa) in insect cells. Both proteins have C-terminal 6xHis-tags, and the purified proteins eluted at a similar position. There is no significant shift in SEC elution position in comparison with Gpi16 alone. (**C**) Native PAGE analysis on purified *S. cerevisiae* Gpi16 (20-550aa), *S. cerevisiae* Gpi8 (23-306aa), and the complex formation. (**D**) SEC profile and SDS-PAGE analysis on co-expressed *S. cerevisiae* Gpi16 (20-550aa) and *S. cerevisiae* Gpi8 (23-379aa) in insect cells. Both proteins have C-terminal 6xHis-tags. The purified proteins eluted out at different positions, indicating lack of complex formation. (**E**) Non-reducing SDS-PAGE followed by anti-FLAG Western blotting for purified GPIT from the *S. cerevisiae* strain expressing Gpi8-3xFLAG. (**F**) Non-reducing SDS-PAGE followed by anti-FLAG Western blotting for purified GPIT from *S. cerevisiae* strain expressing Gpi16-3x FLAG.

We wondered if *S. cerevisiae* Gpi8 and *S. cerevisiae* Gpi16 interact differently from the human counterpart. To address this question, we first expressed *S. cerevisiae* Gpi16 luminal domain (20-550aa) as a secreted protein in insect cells. Since *S. cerevisiae* Gpi16 contains 8 Cys residues likely with complicated disulfide pairings, we did not attempt expression in *E. coli*. Insect cell expression of Gpi16 resulted in a good yield of the secreted protein. The elution position in the SEC profile corresponded to the molecular mass of monomeric Gpi16 (Fig. 6A).

Having obtained purified proteins of both *S. cerevisiae* Gpi8 and *S. cerevisiae* Gpi16, we tested whether they form a complex through co-expression in insect cells. We reasoned that the proper redox environment in the secretory pathway could be essential for formation of the disulfide-linked Gpi8-Gpi16 dimer, if they behave similarly to the case for human GPIT. We co-expressed Gpi16 with either Gpi8 caspase-like domain (23-306aa) or the Gpi8 full-length luminal domain (23-379aa). Both Gpi16 and Gpi8 constructs are His-tagged and both proteins were purified together from Ni-affinity chromatography. However, the SEC profiles and native PAGE of the co-expressed proteins showed that neither Gpi8-Gpi16 pairs co-migrated (Fig. 6B–D). Because dimeric Gpi8 (23-306aa) has a similar molecular mass as monomeric Gpi16, their elution positions in the co-purified mixture are similar (Fig. 6B). Lack of interaction was nonetheless clearly seen on native PAGE of the gel filtration peak, in which Gpi16 and Gpi8 migrated separately as individual bands without generation of any band shift due to complex formation (Fig. 6C). Similarly, individually purified Gpi8 (23-306) and Gpi16 also did not form a complex on native PAGE (Fig. 6C).

There may be at least two reasons why *S. cerevisiae* Gpi8 and *S. cerevisiae* Gpi16 failed to form a complex despite the predicted disulfide bond linkage. One possibility is that Gpi8 and Gpi16 only form a complex in the context of the entire five-subunit GPIT complex. Another possibility is that the *S. cerevisiae* GPIT is somewhat different from the human GPIT in disulfide pairing, as not all Cys residues are conserved (Fig. 2A). To elucidate whether Gpi8 and Gpi16 are disulfide linked in the *S. cerevisiae* GPIT complex, we purified endogenous GPIT complexes using the engineered strains containing 3xFLAG tag on either Gpi8 or Gpi16, similarly as what we did in Fig. 1. To preserve potential inter-chain disulfide pairings, we used non-reducing SDS-PAGE followed by anti-FLAG Western blot (Fig. 6E,F). Anti-FLAG blots on the Gpi8-3xFLAG GPIT complex and the Gpi16-3xFLAG GPIT complex showed major bands at close to the expected molecular weights of Gpi8 and Gpi16, respectively. Both Gpi8 and Gpi16 run somewhat slower than the expected monomeric molecular weights likely due to the addition of the extended 3xFLAG tag. Unlike the case for human GPIT^15^, there are no major higher molecular weight bands that could correspond to the disulfide linked *S. cerevisiae* Gpi8-Gpi16 subunits. The minor bands in both anti-FLAG blots for Gpi8 and Gpi16 are above the 260 kDa marker and therefore unlikely correspond to the Gpi8-Gpi16 complex.

## Discussion

GPIT is an important enzyme complex that has been associated with human genetic diseases such as paroxysmal nocturnal haemoglobinuria (PNH)^16^ and other intellectual disability syndromes^17^. In plant, the catalytic subunit Gpi8 of GPIT plays an essential role in stomata formation and fertility^26^. Here, we report an initial attempt to characterize *S. cerevisiae* GPIT by purification of the endogenous complex and by recombinant expression. Our findings suggest a dimeric GPIT complex, likely mediated by the Gpi8 subunit, which shows similarities to caspases. This Gpi8-mediated dimerization appears to be stabilized by disulfide bond formation and by glycosylation. The dimeric GPIT supposedly provides a large surface for all the transamidation reaction steps. This scenario is different from the previously reported data on human GPIT in which Gpi16 and Gpi8 stabilize each other by a disulfide bond^15^ and exist in the ternary complex of Gaa1, Gpi8, and Gpi16^6^, which may be important for future structural studies on this complex.

## Materials and Methods

### Purification of Endogenous Yeast GPIT and Blue Native PAGE Assay

GPIT was separately purified from each of four different *S. cerevisiae (S. cerevisiae*) strains containing 3xFLAG-Gpi8, 3xFLAG-Gaa1, 3xFLAG-Gpi16, or 3xFLAG-Gpi17 using the following steps. The yeast cells were cultured in YPD medium at 37 °C until ~9,000 OD_600_ units of yeast (~2.4 liter culture at a cell density of OD_600_ ~3.7). The yeast cells then were lysed using glass beads (VWR cat # 101454-156) in disruption buffer containing 5% glycerol, 20 mM HEPES at pH 7.5, 10 mM MgCl_2_ and protease inhibitors (Sigma cat # S8830). After separating glass beads and cell debris at 1,000 × g, microsomal membranes were collected in high speed at 4 °C for 20 min. GPIT extraction was done in 1% digitonin (Sigma cat # D141-500 MG), 20 mM HEPES pH = 7.5, 100 mM NaCl and protease inhibitors for 60 min on ice. Collected digitonin extract of microsomes were pre-cleared for non-specific binding using IgG beads in buffer containing 0.1% digitonin, 20 mM HEPES at pH 7.5, 100 mM NaCl and protease inhibitors before being treated with anti-FLAG agarose. The bound yeast GPIT complexes were eluted with 5 mg/mL 3xFLAG at 4 °C with shaking for 2 hour, and the samples were loaded either on a 10% SDS-PAGE gel followed by colloidal Coomassie Blue staining or on a freshly made 6% Blue native PAGE to determine native protein masses and oligomeric states. We casted the Blue native PAGE following the published protocol^21^.

### Cloning and Protein Expression in *E. coli*

Bacterial expression of *S. cerevisiae* Gpi8 (23-306aa)^BL21^, *S. cerevisiae* Gpi8 (23-339aa)^BL21^, *S. cerevisiae* Gpi8 (23-359aa)^BL21^, *S. cerevisiae* Gpi8 (23-379aa)^BL21^, *S. cerevisiae* Gpi8 (23-306aa)^Cys85Ala^, *S. cerevisiae* Gpi8 (23-306aa)^Cys199Ala^ and *S. cerevisiae* Gpi8 (23-306aa)^Cys244Ala^ used the following cloning and expression methods. The luminal portion of the *Saccharomyces cerevisiae* Gpi8 sequence (*S. cerevisiae* Gpi8; NCBI RefSeq: NM_001180639.1) was used as a template for PCR reactions, and each PCR product was sub-cloned into NdeI-XhoI sites of pET26a (Novagen). The *S. cerevisiae* Gpi8 constructs were transformed into *E. coli* BL21-CodonPlus^®^ (DE3)-RIPL competent cells (Agilent Technologies) and *E. coli* Rosetta-gami 2 (DE3) pLysS (Novagen) for cytosolic protein expression. The *E. coli* containing the expression vectors were inoculated in Luria-Bertoni (LB) broth containing 100 μg/ml kanamycin and 100 μg/ml chloramphenicol until an OD_600_ of 0.6 was reached, and isopropyl β-D-thiogalactoside (IPTG) was added to a final concentration of 1 mM. The culture was allowed to grow over night at 20 °C before harvesting at 4,000 × g for 15 min.

### Cloning and Expression of Secreted Proteins in Insect Cells

Expressing *gpi8 and gpi16* genes as secreted proteins in insect cells may be necessary to promote proper folding and disulfide bond formation. The luminal portions of *S. cerevisiae* Gpi8 (UniParc: P49018) and *S. cerevisiae* Gpi16 (UniParc: P38875) were inserted into the baculovirus pFastBac-dual vector (Invitrogen) with an N-terminal gp67 signal peptide, which is an effective signal sequence for protein secretion, and a C-terminal His-tag. Each pFastBac-dual construct was first transformed into DH10Bac *E. coli* for transposition into the bacmid, and the isolated bacmid DNA was transfected in *Spodoptera frugiperda* Sf9 insect cells using Cellfectin II Reagent. Once virus plaques developed, the plaques with recombinant viruses were picked and expanded. The virus stock was then used to infect a fresh culture of insect cells, resulting in expression of Gpi8 or Gpi16 proteins. To inhibit glycosylation of insect cell-expressed secreted proteins, 2.5 mg/L or 5 mg/L tunicamycin (Sigma-Aldrich) was added into the medium during insect cells infection.

### Protein Purification

Both bacterial expressed and insect cell-expressed proteins were purified using Ni-affinity chromatography and size exclusion chromatography (Superdex 200 10/300 GL). The bacteria cells were lysed on ice by sonication in Buffer A (50 mM Tris-HCl pH 8, 300 mM NaCl and 15 mM imidazole), added with 5 mM β-Mercaptoethanol if purifying proteins under a reducing condition. The supernatant that was separated from the *E. coli* lysate through centrifugation was incubated with Ni-NTA beads (GE Healthcare) for one hour at 4 °C with rotation. The insect cell medium that contained the expressed secreted protein was loaded onto a pre-packed Ni column directly after pH adjustment. The Ni-NTA beads bound with the protein were washed 3 times with Buffer B (50 mM Tris-HCl at pH 8, 300 mM NaCl, 35 mM imidazole, with or without 5 mM β-mercaptoethanol). The proteins were eluted with Buffer C (50 mM Tris-HCl at pH 8, 300 mM NaCl, 150 mM imidazole, with or without 5 mM β-mercaptoethanol). Subsequently, the concentrated protein eluent was subjected to size exclusion chromatography using Buffer D (20 mM Tris-HCl at pH 8 and 150 mM NaCl, with or without 2.5 mM DTT).

### Non-reducing SDS-PAGE

To capture disulfide-linked species, we used an SDS sample buffer without any reducing agent. The proteins were mixed with the sample buffer and heated at 95 °C for 5 minute before being loaded onto SDS-PAGE gels.

### Structural Modeling

Because Gpi8 has been predicted to have a caspase-like fold, we used the full-chain protein structure prediction server Robetta^25^ to model the structure of its luminal domain. We generated five independent structural models, and the caspase-like core domain models are highly similar to each other. The non-core region C-terminal to the caspase-like domain preferentially forms a structure with a central three-stranded β-sheet flanked by α-helices.

### Small-angle X-ray Scattering (SAXS) Experiments and Data Analysis

The small-angle X-ray scattering data of the various Gpi8 recombinant proteins were collected using the X9 beamline at the National Synchrotron Light Source of the Brookhaven National Laboratory. The purified proteins were stored in Buffer D containing 20 mM Tris-HCl at pH 8.0 and 150 mM NaCl before SAXS experiments. Each blank or sample was measured in triplicate, and the sample measurements were adjusted by subtracting the scattering from buffer-alone blank. Several protein concentrations (2.5 to 7.0 mg/ml) were used for each recombinant protein, in order to assess and remove any concentration-dependent inter-particle effects. All data were analyzed and processed using program packages PRIMUS^27^, and GNOM^28^. The SAXS envelopes were built using the program RasWin.

## Additional Information

**How to cite this article**: Yi, L. *et al*. Disulfide Bond Formation and *N*-Glycosylation Modulate Protein-Protein Interactions in GPI-Transamidase (GPIT). *Sci. Rep.* **7**, 45912; doi: 10.1038/srep45912 (2017).

**Publisher's note:** Springer Nature remains neutral with regard to jurisdictional claims in published maps and institutional affiliations.

## Supplementary Material

Supplementary Information
