# Supplementary material for: Disulfide Bond Formation and N-Glycosylation Modulate Protein-Protein Interactions in GPI-Transamidase (GPIT)
Source: Sci Rep. 2017 Apr 4;7:45912. doi: 10.1038/srep45912 (PMC5379210; doi:10.1038/srep45912)
Supplement: Supplementary Information [file srep45912-s1.pdf]

Supplementary Information:

Disulfide Bond Formation and *N*-Glycosylation Modulate Protein-Protein Interactions in GPI-Transamidase (GPIT)

Lina Yi[1,2,3], Gunes Bozkurt[1,2], Qiubai Li[1,2], Stanley Lo[4, 5], Anant K. Menon[4] and Hao Wu[1,2,3,*]

[1]Department of Biological Chemistry and Molecular Pharmacology, Harvard Medical School, Boston, MA 02115
[2]Program in Cellular and Molecular Medicine, Boston Children's Hospital, Boston, MA 02115
[3]Weill Cornell Graduate School of Medical Sciences, New York, NY 10065
[4]Department of Biochemistry, Weill Cornell Medical College, New York, NY, 10065
[5]Current address: Section of Cell and Developmental Biology and Program in Mathematics and Science Education, University of California San Diego, La Jolla CA 92093

[*]Correspondence to

Hao Wu, Ph.D.
Email address: hao.wu@childrens.harvard.edu
Phone: +1-617-713-8160
Fax: +1-617-713-8161

Supplementary Figures:

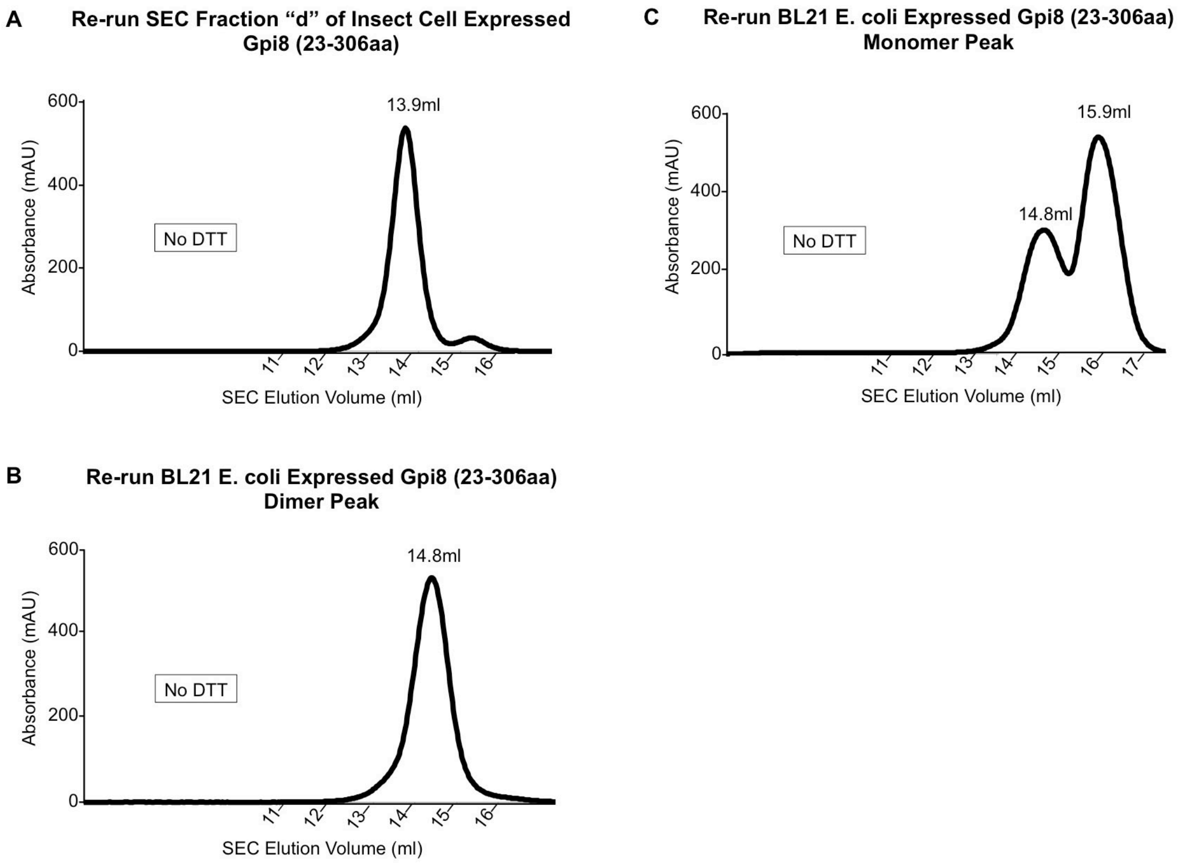

Figure S1. **Re-run of SEC peaks of the *S. cerevisiae* Gpi8 (23-306aa) recombinant proteins.** (A) Re-run of the dimeric SEC fraction from Figure 3A of insect cell-expressed Gpi8 (23-306aa). (B) Re-run of BL21 *E. coli* expressed Gpi8 (23-306aa) dimer peak from Figure 3C. (C) Re-run of BL21 *E. coli* expressed Gpi8 (23-306aa) monomer peak from Figure 3C.

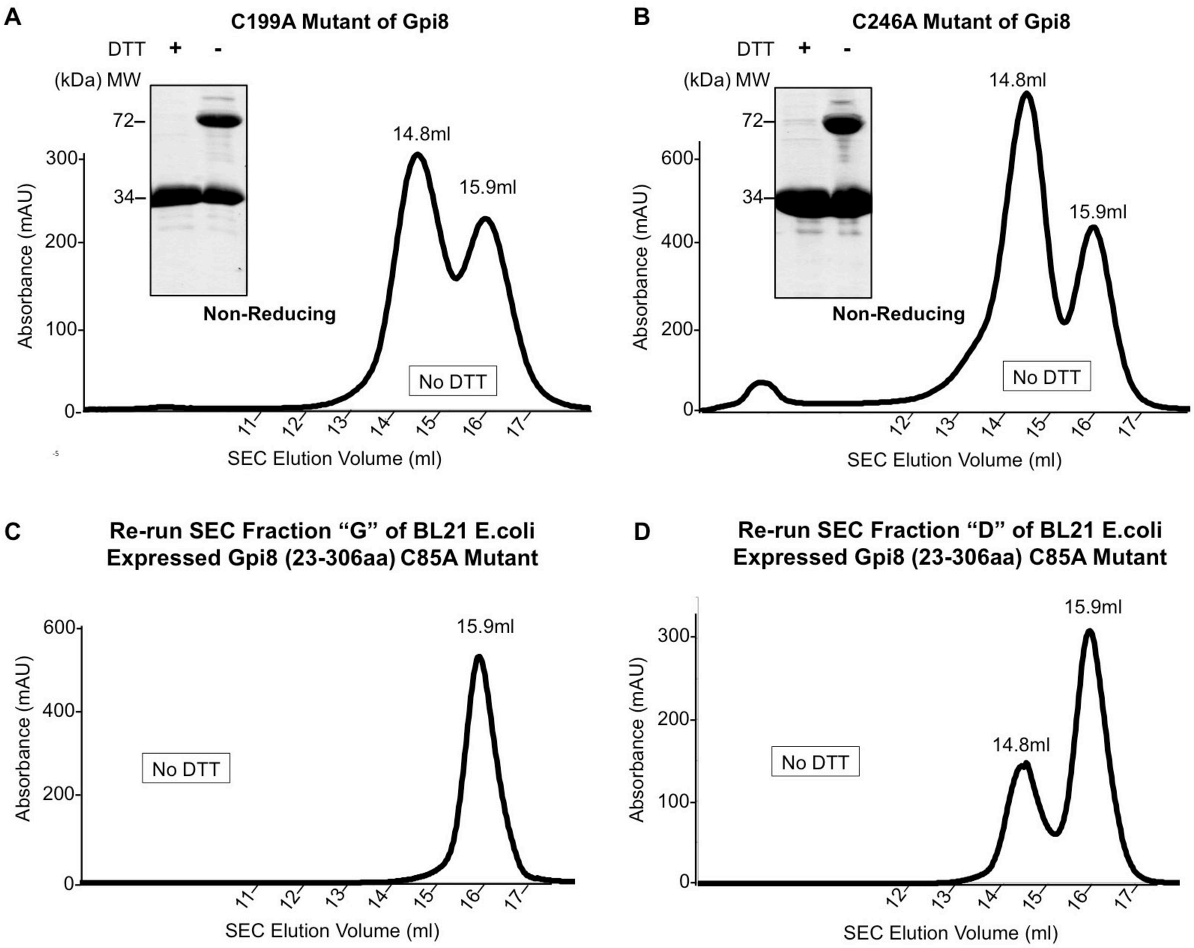

Figure S2. ***S. cerevisiae*** **Gpi8 caspase-like domain form homo-dimers through residue Cys85.** (A) SEC profile and SDS-PAGE of *E. coli* BL21 expressed Gpi8 (23-306aa) C199A mutant. (B) SEC profile and SDS-PAGE of *E. coli* BL21 expressed Gpi8 (23-306aa) C246A mutant. (C) SEC profile and SDS-PAGE of *E. coli* BL21 expressed Gpi8 (23-306aa) C85A mutant. (D) Re-run of SEC fraction “G” monomer of the Gpi8 (23-306aa) C85A mutant from Figure 4B. (E) Re-run of SEC fraction “D” dimer of the Gpi8 (23-306aa) C85A mutant from Figure 4B.

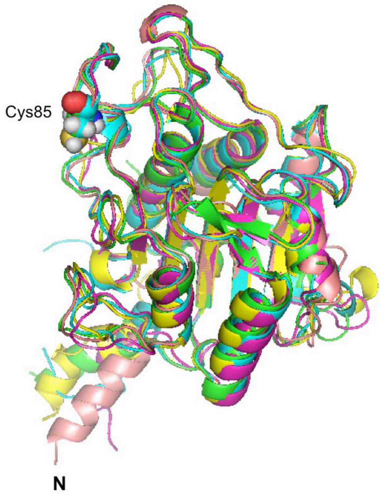

Figure S3. Alignment of five Robetta structural models of the *S. cerevisiae* Gpi8 (23-306aa) caspase-like domain
